# A support vector machine-based tool for rapid pediatric asthma exacerbation risk assessment: development and nursing application

**DOI:** 10.3389/fped.2025.1660895

**Published:** 2025-10-07

**Authors:** Hui Tang, Guihong Yang, Xudan Gu, Haiyan Mao, Huling Cao

**Affiliations:** ^1^ Department of Pediatrics, The First People’s Hospital of Nantong (Second Affiliated Hospital of Nantong University), Nantong, China; ^2^ School of Nursing and Rehabilitation, Nantong University, Nantong, China

**Keywords:** pediatric asthma, support vector machine, asthma risk assessment, WeChat mini-program, nursing application

## Abstract

**Background:**

Childhood asthma poses a significant threat to pediatric health, and traditional assessment methods are often inadequate in efficiency and accuracy. This study aims to develop a rapid assessment tool for pediatric asthma exacerbation risk based on the support vector machine (SVM) algorithm and evaluate its value in nursing practice.

**Methods:**

Clinical data from children with asthma were collected, incorporating key indicators including eczema, allergic rhinitis (AR), family medical history (FMH), dyspnea, white blood cell count (WBC), immunoglobulin E (IgE), and fractional exhaled nitric oxide (FeNO). An SVM-based risk prediction model was developed. Utilizing Plumber, an application programming interface (API) was constructed to enable data transmission and real-time risk assessment. The pediatric asthma risk rapid tool (PARRT) mini-program was subsequently developed. Service quality metrics were compared before and after PARRT implementation.

**Results:**

The constructed SVM model demonstrated excellent performance on the test dataset, achieving an area under the curve (AUC) of 0.9998. Clinical application revealed that PARRT significantly reduced patient wait time, decreased report wait time, improved satisfaction scores among patients and their families, as well as enhanced nursing staff efficiency.

**Conclusion:**

PARRT exhibits strong predictive accuracy and holds considerable promise for clinical utility in pediatric asthma management.

## 1 Introduction

Asthma stands as one of the most prevalent chronic respiratory diseases globally in children, significantly compromising their health and quality of life (1). Data from the World Health Organization indicate that children constitute over half of all asthma patients worldwide, with the incidence rate demonstrating a persistent upward trend (2). Currently, the assessment of asthma exacerbation risk in children relies primarily on conventional methods, including clinical symptoms, pulmonary function tests, serological markers such as immunoglobulin E (IgE) levels, and medical history review (3, 4). However, these approaches exhibit significant limitations, creating a pressing clinical need for the development of a rapid and objective risk assessment tool applicable to children across various age groups.

Machine learning has become a popular method for developing predictive models in the medical field due to its ability to process complex and massive amounts of health data (5). In recent years, more and more machine learning (ML)—based prediction models have been developed to predict asthma attacks (6, 7), with supervised learning as the main approach, such as LASSO logistic regression, random forest, etc (6). Among them, support vector machines have attracted attention due to their small sample generalization ability.

Support vector machine (SVM), a classical algorithm in machine learning, has demonstrated superior classification and predictive performance when handling small-sample and high-dimensional data (8). In recent years, SVM has been successfully applied in diverse medical fields, such as cardiovascular disease risk prediction (9) and the diagnosis of diabetic complications (10). Its core mechanism, which constructs an optimal hyperplane in a high-dimensional space to differentiate distinct classes of data, aligns well with the requirement for multi-parameter integrated analysis inherent in asthma exacerbation risk assessment (11). By uncovering potential associations between multi-dimensional clinical data and asthma exacerbation risk, SVM holds promise for overcoming the bottlenecks of traditional methods, enabling a precise quantitative assessment of exacerbation risk in pediatric asthma.

This study aims to leverage the SVM algorithm to integrate multi-dimensional clinical data, thereby developing a rapid assessment tool for pediatric asthma exacerbation risk and exploring its potential clinical utility in nursing practice. Beyond providing healthcare professionals with an objective and efficient assessment method to aid in the early identification of high-risk children and optimize nursing intervention strategies, the successful development of this tool is anticipated to offer a novel technological pathway for the precision management of childhood asthma. Ultimately, this approach seeks to improve disease control and enhance the quality of life for affected children.

## 2 Materials and methods

### 2.1 Study participants

This retrospective study enrolled 103 children with asthma who were treated in the Department of Respiratory Medicine of our hospital between January 2021 and December 2023. Using a random number table method, 103 healthy children undergoing routine health examinations in our hospital during the corresponding period were selected as the control group. Additionally, a separate cohort of 43 pediatric asthma patients treated in the Department of Respiratory Medicine of our hospital between February 2024 and August 2024 was recruited for pre-post validation analysis. This study was approved by the Medical Ethics Committee of Second Affiliated Hospital of Nantong University (2023KT236). Demographic and baseline characteristics demonstrated no significant differences between the main study groups, rendering them eligible for subsequent comparative analysis (Table 1).

Retrospective asthma group: 103 children with confirmed asthma (January 2021–December 2023, Department of Respiratory Medicine);
Retrospective control group: 103 healthy children (matched via 1:1 propensity score matching with the asthma group, same period, routine health examinations);
Prospective mini—program validation cohort: 43 children with confirmed asthma (February 2024–August 2024, for pre—post workflow evaluation, not overlapping with the retrospective cohort).

**Table 1:** Comparison of clinical characteristics between asthma and control groups.

Variable	Category	Control group (*n* = 103)	Asthma group (*n* = 103)	*t/X* ^2^	*P*
Gender	Male	57 (55.34)	51 (49.51)	0.701	0.403
	Female	46 (44.66)	52 (50.49)		
Eczema	Yes	20 (19.42)	61 (59.22)	34.201	<0.001
	No	83 (80.58)	42 (40.78)		
AR	Yes	18 (17.48)	83 (80.58)	82.070	<0.001
	No	85 (82.52)	20 (19.42)		
Food allergy	Yes	32 (31.07)	37 (35.92)	0.545	0.46
	No	71 (68.93)	66 (64.08)		
Drug allergy	Yes	16 (15.53)	13 (12.62)	0.361	0.548
	No	87 (84.47)	90 (87.38)		
FMH	Yes	15 (14.56)	75 (72.82)	71.034	<0.001
	No	88 (85.44)	28 (27.18)		
Dyspnea	Yes	17 (16.5)	91 (88.35)	106.581	<0.001
	No	86 (83.5)	12 (11.65)		
Age		7.92 ± 3.33	8.21 ± 3.58	0.586	0.558
WBC (10^9^/L)		8.18 ± 4.34	11.43 ± 2.66	−6.491	<0.001
IgE (IU/ml)		67.35 ± 15.72	119.32 ± 22.20	−19.391	<0.001
PET (L/min)		331.69 ± 77.39	344.52 ± 76.47	−1.197	0.233
FeNO (ppb)		14.85 ± 6.32	24.62 ± 5.83	−11.533	<0.001

AR, allergic rhinitis; FMH, family medical history; WBC, white blood cell count; IgE, immunoglobulin E; PEF, peak expiratory flow; FeNO, fractional exhaled nitric oxide.

The table only presents baseline data of the “retrospective asthma group (*n* = 103)” and “retrospective control group (*n* = 103)”.

Inclusion criteria were as follows: (1) Children aged 2–17 years were eligible for inclusion (12). (2) Participants in the asthma group were required to meet the diagnostic criteria for childhood asthma as defined by the Diagnosis and Management of Asthma in Children guidelines (13). (3) All participants were required to have complete clinical records available (4). Written informed consent was obtained from both the participating children and their parents or legal guardians. Exclusion criteria encompassed: (1) the presence of other respiratory diseases such as interstitial lung disease or congenital airway malformations; (2) significant comorbidities including severe cardiac, hepatic, renal, or systemic diseases, immunodeficiency disorders, or severe infections; (3) concurrent psychiatric or cognitive impairments; and (4) loss to follow-up during the study period.

### 2.2 Data collection

In the retrospective study of the first stage, for both the asthma and control groups, data including documented histories of eczema, allergic rhinitis (AR), food allergies, drug allergies, and dyspnea, as well as family medical history (FMH) were systematically collected. Concurrently, relevant clinical parameters were obtained: white blood cell count (WBC), total IgE levels, peak expiratory flow (PEF), and fractional exhaled nitric oxide (FeNO).

In the prospective study of the second phase, specific nursing and process-related indicators were also recorded. These encompassed (1) patient wait time (PWT), defined as the duration from registration completion to the initiation of the physician consultation; (2) patient and family satisfaction scores (SSs), assessed via a dedicated mini-program using a 5-point Likert scale (1 = very dissatisfied, 2 = dissatisfied, 3 = neutral, 4 = satisfied, 5 = very satisfied); (3) report wait time (RWT), measured from the commencement of testing to the time the patient received the results; (4) the number of on-time nursing procedures (OTNP); and (5) the number of cases demonstrating diagnostic accuracy (DIO).

### 2.3 Model development

Data pertaining to pediatric asthma indicators underwent comprehensive preprocessing, including cleaning, feature selection, and standardization. Class imbalance in the original dataset (more asthma cases than age-matched healthy controls) was addressed using 1:1 propensity score matching (PSM) with age, gender, and residential area as matching variables, resulting in a final balanced dataset of 103 asthma cases and 103 healthy controls.Class imbalance within the dataset was addressed using appropriate techniques. The radial basis function (RBF) kernel function was selected for the SVM model. Hyperparameter optimization, specifically for the penalty parameter C and the kernel parameter *γ*, was performed via grid search coupled with 10-fold cross-validation utilizing the training dataset. This process aimed to identify the optimal hyperplane maximizing the margin between classes. The resulting model, fitted on the training data, was subsequently evaluated using an independent test set. Model performance was rigorously assessed using established metrics, including accuracy, the area under the receiver operating characteristic (ROC) curve (AUC), and the F1 score. The clinical utility and net benefit of the model were further evaluated using decision curve analysis (DCA). To enhance interpretability, feature importance rankings were generated to elucidate the model's decision logic. The classification threshold was then calibrated based on specific clinical requirements to optimize predictive utility. A mechanism for periodic data updates and model retraining was implemented to ensure sustained performance. The overarching goal was to develop a quantifiable predictive tool for assessing pediatric asthma exacerbation risk within nursing practice. All computational procedures were executed using R software. The following R packages were employed for specific tasks: e1071 for SVM model construction, caret for model evaluation and hyperparameter tuning, ggplot2 for data visualization, dplyr for data manipulation, pROC for generating ROC curves, and randomForest for computing feature importance measures.

### 2.4 Pediatric asthma risk rapid tool (PARRT) mini-program development

Following the completion of SVM model training within the R environment, the finalized model object and associated data preprocessing steps were saved as files. To operationalize the model for real-time prediction, an application programming interface (API) was defined using the Plumber package. This API handles incoming hypertext transfer protocol (HTTP) requests and returns the corresponding risk prediction outputs. The Plumber API service was subsequently deployed and hosted on a dedicated server. Upon successful backend API deployment, the front-end user interface for the PARRT was developed as a WeChat mini-program. This involved designing an intuitive form-based interface within the mini-program environment and implementing the functionality to securely call the remote prediction API, transmit user-input data, and display the generated risk assessment results to the end-user.

### 2.5 Statistical analysis

Data processing was performed using SPSS software (version 27.0) and R software (version 4.4.3). Continuous variables conforming to a normal distribution were presented as mean ± standard deviation $\left(\bar{x}\pm s\right)$, while categorical data were expressed as *n* (%). Comparative analyses between groups were conducted using the independent samples *t*-test and the chi-square (*χ*^2^) test. Data visualization was generated using the ggplot2 package within the R environment. A *P* value of less than 0.05 was considered statistically significant.

## 3 Results

### 3.1 Clinical characteristics

Independent samples *t*-tests comparing the clinical characteristics of the asthma group and the control group revealed statistically significant differences (*P* < 0.05) in several parameters (Table 1). Specifically, significant intergroup differences were observed in the prevalence of eczema, AR, FMH, and dyspnea, as well as in the values of WBC, total IgE levels, and FeNO. Leveraging these clinically distinct parameters, we developed a SVM prediction model to assess the risk of acute exacerbations in pediatric asthma.

### 3.2 SVM model construction

**Figure 1:**
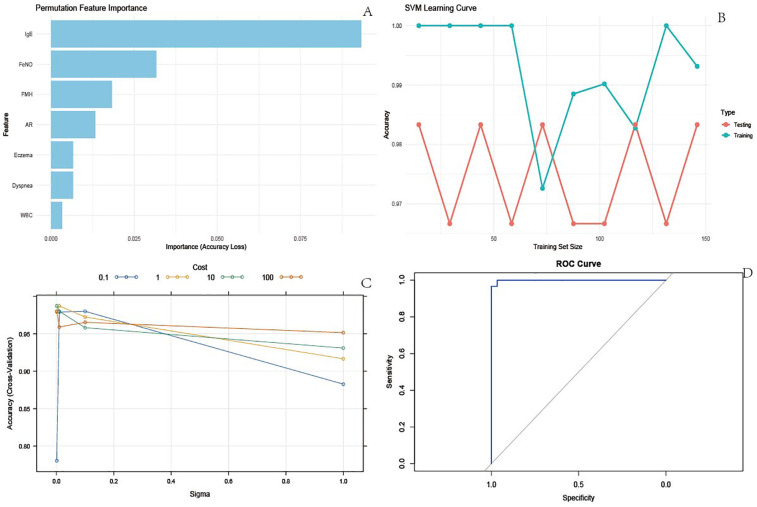
SVM model results. **(A)** Permutation feature importance. Used to measure the contribution of each feature to the accuracy of the model. By randomly arranging the eigenvalues and observing the decrease in model accuracy, the importance of the features can be determined. The longest bar corresponding to IgE indicates that it has the greatest impact on the accuracy of the model and is the most important feature; The bar lengths of features such as FeNO and FMH decrease in order, and their importance also decreases in order; **(B)** SVM learning curve. The learning curve reflects the accuracy changes of the model in the training and testing sets under different training set sizes, and is used to evaluate the overfitting or underfitting of the model; **(C)** SVM parameter tuning results. This demonstrates the accuracy performance of the model in cross validation under different combinations of hyperparameters (Sigma and Cost), used to determine the optimal hyperparameter combination. Different colored lines represent different cost values, with Sigma values on the horizontal axis and Accuracy (Cross Validation) on the vertical axis. It can be seen that there is a specific combination of Sigma and Cost (for example, when Sigma is around 0.1 and Cost is 100), and the cross validation accuracy of the model is high. This set of hyperparameters is the optimal setting used for subsequent model construction, ensuring the best model performance; **(D)** ROC curve. The ROC curve is used to evaluate the performance of binary classification models, with the horizontal axis representing specificity and the vertical axis representing sensitivity. The area under the curve (AUC) is an important evaluation metric.

Clinically significant variables were incorporated into the SVM model. The permutation feature importance method, employed in Figure 1A, assessed the relative contribution of each feature, identifying IgE as the most critical predictor within the SVM framework. Figure 1B depicts the SVM learning curve, indicating stable model performance across both training and test datasets, with performance metrics consistently ranging between 0.96 and 1.00. This stability, coupled with the curve trajectory, demonstrated an absence of overfitting or underfitting. Furthermore, Figure 1C illustrates the results of SVM parameter tuning, showcasing model performance across various parameter combinations. Optimal model fit, characterized by an AUC of 0.9871, was achieved with the hyperparameter values sigma = 0.01 and cost = 1.0. Finally, the ROC curve presented in Figure 1D yielded an AUC of 0.9998, collectively indicating robust overall performance of the SVM model (Figure 1).

### 3.3 SVM decision boundary

**Figure 2:**
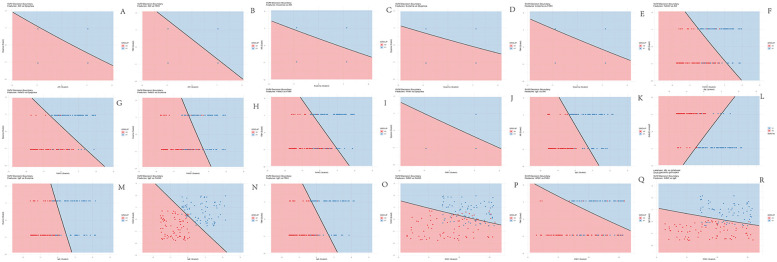
Decision boundary maps of SVM model. **(A)** AR + dyspnea combination; **(B)** AR + FMN combination; **(C)** AR + eczema combination; **(D)** Eczema + dyspnea combination; **(E)** Eczema + FMN combination; **(F)** FeNO + AR combination; **(G)** FeNO + dyspnea combination; **(H)** FeNO + eczema combination; **(I)** FeNO + FMN combination; **(J)** FMN + dyspnea combination; **(K)** IgE + AR combination; **(L)** IgE + dyspnea combination; **(M)** IgE + eczema combination; **(N)** IgE + FeNO combination; **(O)** WBC + FeNO combination; **(P)** WBC + FeNO combination; **(Q)** WBC + FMN combination; **(R)** WBC + IgE combination. X0 = Control group (red); X1 = Asthma group (blue). The solid black line in each map represents the decision boundary learned by the SVM, partitioning the feature space into two distinct regions corresponding to the model's binary risk classification. Greater dispersion of data points across the decision boundary, indicative of clearer separation between the groups, signifies that the respective feature pair contributes more significantly to the model's classification capability.

The decision boundaries of the SVM model visually illustrate the impact of pairwise feature combinations on asthma risk stratification in children, encompassing a total of 18 combinations as detailed in Figure 2. IgE emerged as the most critical feature for the SVM model. As evidenced in Figure 2K, the majority of red data points cluster predominantly on the left side of the boundary, while blue points are concentrated on the right. This spatial distribution indicates that the model classifies subjects with lower IgE levels combined with AR as lower risk, whereas higher IgE levels correspond to a greater propensity for classification into the high-risk category.

### 3.4 Mobile application development

**Figure 3:**
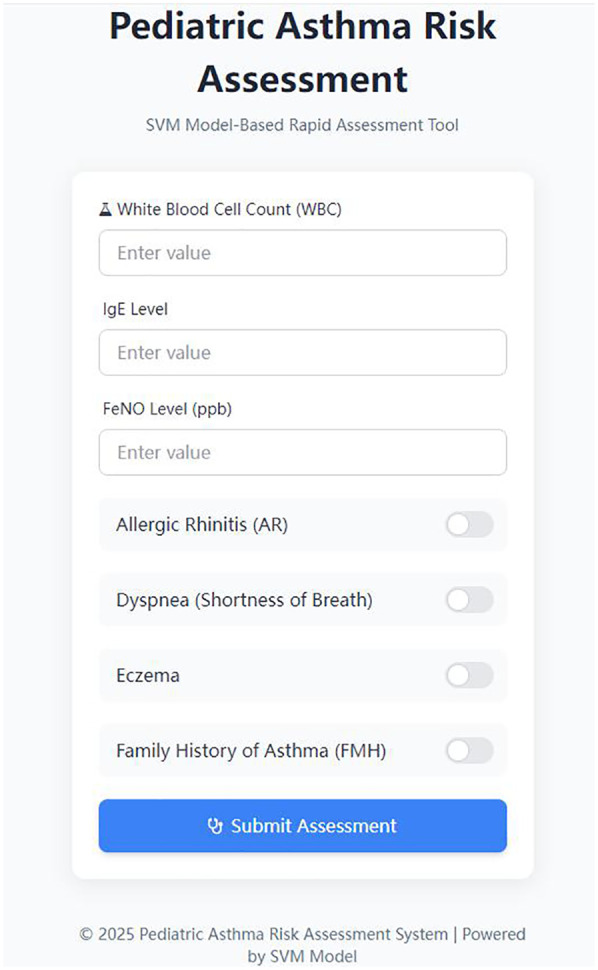
Interface of the pediatric asthma exacerbation risk prediction mobile application.

The PARRT mini-program, developed by our hospital, enabled healthcare providers to input pediatric patients' clinical indicators and receive rapid, real-time predictions of asthma exacerbation risk. Concurrently, patients and their caregivers could promptly access diagnostic results through the application. The three risk levels were determined by integrating the SVM model's probability distribution and clinical intervention needs, with reference to pediatric asthma management guidelines and expert consensus from our hospital's pediatric respiratory team.To enhance clinical utility, risk levels were visually categorized using a color-coded system: green (low risk,SVM probability score <0.3), yellow (moderate risk, SVM probability score between 0.3 and 0.7), and red (high risk), providing an immediate visual alert for medical personnel. Robust security measures were implemented, including application programming interface (API) key authentication to restrict request frequency and prevent malicious calls. Sensitive data (e.g., patient information) were encrypted during transmission for enhanced protection. To optimize performance, the predictive model was cached in server memory, eliminating redundant loading and reducing response latency. Furthermore, input data format underwent dual validation at both the frontend and backend levels. Specific error codes were returned for distinct exception types, and comprehensive API call logs were maintained to facilitate subsequent analysis and troubleshooting. A representative interface of the application is presented in Figure 3.

### 3.5 Mobile application implementation

Following a six-month implementation period of the PARRT mini-program within the hospital setting, an evaluation of its effectiveness was conducted.Among the 43 asthma patients in the prospective cohort, 32 (74.4%) were classified as “high risk” (score >0.7) by the mini-program, and all received timely intervention (e.g., medication adjustment, asthma education)—consistent with their confirmed asthma diagnosis. The results demonstrated that the PARRT mini-program significantly reduced both PWT and RWT, while concurrently improving SSs among patients and their families. These findings are detailed in Table 2.

**Table 2:** Effectiveness evaluation of mobile application implementation.

Item	Category	Pre-implementation (*n* = 43)	Post-implementation (*n* = 43)	*t/X* ^2^	*P*
PWT (min)		23.93 ± 4.26	17.40 ± 4.59	6.843	<0.001
SS		3.98 ± 0.71	4.40 ± 0.49	−3.182	0.002
RWT (min)		72.86 ± 21.89	46.95 ± 16.07	6.256	<0.001
OTNP	On time	40	43	/	0.241
	Delayed	3	0		
DIO	Accurate	42	43	/	1
	In error	1	0		

PWT, patient wait time; SS, satisfaction score; RWT, report wait time; OTNP, on-time nursing procedures; DIO, diagnostic accuracy.

## 4 Discussion

Asthma represents a chronic respiratory disease posing a significant global threat to pediatric health. Recurrent episodes of wheezing and dyspnea not only severely impact children's growth, development, and quality of life but can also lead to irreversible pulmonary impairment and even life-threatening complications (14). Furthermore, frequent acute exacerbations substantially increase the burden of emergency department visits and hospitalizations, imposing considerable economic strain on families and society (15). Notwithstanding these challenges, the limitations inherent in traditional assessment methods often result in suboptimal efficiency and accuracy in risk prediction, failing to adequately meet clinical demands. The present study successfully developed and validated a SVM-based rapid assessment tool for pediatric asthma exacerbation risk. This tool integrates multi-dimensional clinical indicators, offering an innovative solution for precision nursing care in childhood asthma. The advantages and potential challenges demonstrated throughout the entire process, from model construction to clinical implementation, warrant thorough examination.

Regarding model performance and scientific robustness, the SVM algorithm, leveraging its powerful capacity for non-linear classification (16), effectively captured the complex non-linear relationships between key clinical indicators, such as IgE, FeNO, and WBC, and the risk of asthma exacerbation. The model consistently demonstrated high predictive accuracy and AUC values across both training and testing datasets. Eczema and AR, as common allergic conditions, were linked to asthma through the “atopic march”. Studies have indicated (17, 18) that children with eczema or AR exhibit heightened susceptibility to airway inflammation activation and a significantly elevated risk of developing asthma. A FMN of allergic diseases reflects genetic predisposition, and children with an asthmatic family member possess a higher probability of carrying relevant susceptibility genes and are consequently more prone to developing asthma (19). Dyspnea, a hallmark symptom of acute asthma attacks, directly reflects disease control status through its frequency and severity (20). WBC serves as a crucial indicator of systemic inflammation, and its abnormal elevation often signals potential infection, a common trigger for pediatric asthma exacerbations (21). IgE, as the key antibody mediating allergic reactions, demonstrates a close association with the allergic mechanisms underlying asthma, with elevated serum IgE levels signifying a sensitized state (22). Conversely, FeNO functions as a specific biomarker of airway inflammation, with increased levels indicative of eosinophilic airway inflammation. Consequently, FeNO measurement serves as a valuable tool for asthma diagnosis, monitoring disease activity, and assessing therapeutic response (23). Collectively, these indicators comprehensively encompass critical influencing factors across genetic, allergic, inflammatory, and symptomatic dimensions, providing a robust foundation for the model's precise predictive capabilities. Simultaneously, the SVM's utilization of kernel functions to handle high-dimensional data effectively circumvented the “curse of dimensionality”. Within the context of pediatric asthma data characterized by relatively small sample sizes and numerous features, this approach facilitated a more precise delineation of risk boundaries (24). The visualized decision boundary maps offer an intuitive representation of the impact of different feature combinations on risk classification. This visualization provides substantial support for clinical nursing staff in comprehending the model's logic, thereby fostering greater trust in the assessment outcomes and enhancing their utility in guiding practical nursing decisions.

From the perspective of clinical utility, this tool significantly enhances the efficiency and accessibility of pediatric asthma risk assessment. Utilizing an API built between the mobile application frontend and a Plumber backend for data exchange, nursing personnel can input basic patient clinical information and obtain a risk assessment result within a short timeframe. This effectively addresses the limitations of traditional assessments, which often rely on experiential judgment and are time-consuming. In emergency triage scenarios, the rapid identification of high-risk children enables the prioritization of diagnostic and therapeutic resources, thereby expediting critical interventions. During outpatient follow-up, the tool assists nurses in promptly adjusting management strategies, such as intensifying medication guidance and environmental control recommendations for high-risk individuals. Such targeted interventions hold promise for reducing asthma exacerbation frequency and improving the quality of life for affected children. Furthermore, the tool's generation of visually intuitive risk level prompts (e.g., green/yellow/red indicators) allows nursing staff to quickly identify patients requiring heightened attention, optimizing the allocation of nursing resources and enhancing overall service efficiency.

In summary, this study successfully constructed the PARRT and implemented it within a mobile application framework, demonstrably improving hospital-wide nursing service efficiency. Notwithstanding these achievements, several limitations merit consideration. Primarily, the model was trained predominantly on data derived from a single center, introducing potential constraints related to the geographical and ethnic distribution of the sample. This limitation may restrict the model's generalizability to broader populations. Future research necessitates expanding the sample inclusion criteria and integrating multi-center data to further validate the model's applicability across diverse demographic groups. Secondly, although several key clinical indicators were incorporated, the pathogenesis of childhood asthma is inherently complex, influenced by a multitude of genetic, environmental, and psychological factors. Certain potential determinants remain unaccounted for in the current model. Subsequent investigations could explore the integration of more diverse data sources, such as genetic testing results and detailed environmental exposure questionnaires, to refine the model architecture and enhance predictive accuracy. Finally, regarding practical clinical deployment, the widespread adoption of the tool may encounter challenges related to nursing staff training requirements and system compatibility issues. Addressing these will necessitate the development of comprehensive training programs and enhanced integration capabilities with existing hospital information systems.

## Data Availability

The original contributions presented in the study are included in the article/Supplementary Material, further inquiries can be directed to the corresponding author.
